# Enhanced Accuracy in Jump Power Estimation Using Photoelectric Cell System and GRS80 Location-Specific Gravitational Acceleration

**DOI:** 10.3390/s25165163

**Published:** 2025-08-20

**Authors:** J. L. González-Montesinos, F. G. Montesinos, J. R. Fernández Santos, A. Suárez Llorens, I. Caraballo, P. Gutiérrez-Mulas, J. V. Gutiérrez-Manzanedo

**Affiliations:** 1 Department of Physical Education, Faculty of Education Sciences, University of Cádiz. 11510 Cádiz, Spain; jgmontesinos@uca.es (J.L.G.-M.); jorgedelrosario.fernandez@uca.es (J.R.F.S.); josegu.manzanedo@uca.es (J.V.G.-M.); 2 Faculty of Mathematics Sciences, Complutense University of Madrid, 28040 Madrid, Spain; fuensant@ucm.es; 3 Research Group “Geodesia”, Complutense University of Madrid, 28040 Madrid, Spain; 4 Department of Statistics and Operation Research, University of Cádiz, 11510 Cádiz, Spain; alfonso.suarez@uca.es; 5 Faculty of Physical Activity and Sport Sciences, Complutense University of Madrid, 28040 Madrid, Spain; palomagutierrezmulas@gmail.com

**Keywords:** accumulated error, Geodetic Reference System, muscle power, vertical jump

## Abstract

**Highlights:**

**What are the main findings?**

- Using the standard gravity value (*g* = 9.81 m·s^−2^) reduces the accuracy of jump power calculations performed with photoelectric cell systems.
- Significant differences in calculated jump power are observed when using the standard gravity value instead of the local GRS80 gravity. Specifically, power is underestimated at high-altitude locations and overestimated at lower altitudes.

**What is the implication of the main finding?**

- The local value of gravity should be taken into account to enhance accuracy and reduce errors in jump power measurements. Using GRS80-derived local gravity values improves the precision of these calculations.
- It is recommended that devices relying on the gravitational value (*g*) incorporate local gravity values from the GRS80 model into their software to improve the accuracy of sports performance assessments.

**Abstract:**

Power is essential in sports and is typically calculated using a standard gravity value of *g* = 9.81 m·s^−2^. However, this value varies according to altitude and geographical latitude. The aim of this study was to improve the accuracy of power calculations using a photoelectric cell system and the local g value. First, the uncertainty in jump power calculation induced by the direct measurements involved in its estimation was analyzed in this cross-sectional study. Subsequently, the power values obtained for ten volleyball players were calculated through repeated jump tests of 15, 30, and 60 s, using a kinematic system composed of a transmitting bar and a receiving bar with 96 infrared LEDs that detect flight and ground times for each jump. The local gravity values for 34 different locations—obtained through the Geodetic Reference System, taking into account the altitude of each location—and the standard value of *g* = 9.81 m·s^−2^ were used for the power calculation. Significant differences were observed, with underestimation occurring at higher altitude locations and overestimation at lower altitudes. To conclude, the results indicated that the geographic location of the experiment should be considered, and the use of GRS80 local gravity values is recommended to improve the accuracy of jump power calculations.

## 1. Introduction

Many sports require the ability to generate force in a short period of time. This capacity is considered a key performance characteristic for achieving success in most sports modalities [1]. It is a fact that the force of gravity significantly influences performance-related skills in different sports disciplines. Jumping is one of the most widely used skills in sports such as basketball [2] and volleyball [3] and in athletics disciplines such as the high jump, long jump, and throwing. Both the maximum distance obtained when jumping and the ability to perform multiple repeated jumps are highly related to the ability of the athlete’s lower limbs to generate force.

At the 1968 Mexico Olympics, scientists and coaches faced an expected decline in the performance of endurance athletes and established optimal preparation and altitude acclimatization programs for competing at altitude [4]. However, a decrease in performance was not expected in disciplines that do not require high aerobic capacity, where muscle power is more decisive; on the contrary, notable improvements due to altitude were anticipated. Examples are the records obtained in the long jump and triple jump.

Some authors [5] suggested that the high altitude of Mexico City (2260 m), which represents lower gravity values than other locations such as Tokyo (Japan), with a lower altitude (14 m) and different latitude, could aid in achieving these records.

Linthorne (2016) [6] stated that a fair system of recognizing athletics records should consider the influence of environmental conditions on performance. The study goal was to determine the effect of Mexico City’s altitude on the time for a 100 m sprint compared to the same sprint at sea level. The observed time advantage was 0.19 s (±0.02) for men and 0.21 s (±0.05) for women. Therefore, the altitude of the competition venue, as well as the wind speed during the race, should be taken into account when recognizing record performances.

Power measurement and gravity value:

In laboratory settings, vertical jumps are often used as a measure of physical performance for calculating power and anaerobic capacity [7]. Performance is usually quantified using instruments such as force platforms [8], kinematic systems with high-speed cameras [9], systems with optical synchronization [10], and photoelectric cell systems, such as OptoJump [8,11].

The height reached and power developed can be quantified via formulas derived from the linear kinematics of falling bodies by the following expression:

(1) $$H_{v}=\frac{t_{v}^{2}\cdot g}{8}$$

*H_v_: Vertical jump height (m).*

*t_v_: Time the subject remains in the air after take-off (s).*

*g: Gravity value (m·s^−2^).*

The Reactive Jump (RJ) test is commonly employed to measure total power output (P). This test is one of the most widely used methods for quantifying lower-body muscular power and assessing the degree of neuromuscular fatigue induced by intense physical effort [12]. To this end, the subject must perform repeated maximum-intensity vertical jumps for a predetermined duration (i.e., 15, 30, or 60 s), minimizing ground contact time between jumps and maintaining a consistent knee flexion angle of 90° during each jump [13].

The total power developed (*P*) per body mass in the execution of an RJ test is calculated via the expression [14]:

(2) $$P=\frac{g^{2}\cdot t_{v}\cdot t_{t}}{4\cdot t_{c}}$$

*P:* Power (W).

*t_v_:* Time the subject is in the air during the jump (s).

*t_c_:* Time the subject is in contact with the ground (s).

*t_t_:* Total test time (s).

*g:* Gravity value (m·s^−2^).

To calculate the average power $\bar{P}$, it suffices to divide Formula (2) by the number of jumps (*n*) performed during the test execution.

Therefore, to calculate the power exerted, we need to know the value of acceleration caused by gravity exerted by the Earth, among other data. This value has usually been considered as a constant, with an average value of *g* = 9.81 m·s^−2^ (9.80665 m·s^−2^). Standard gravity has been used since 1901, when it was established at the 3rd General Conference on Weights and Measures [15]. The mean value of gravitational acceleration has traditionally been employed to simplify calculations in the majority of physics problems. Even with current technological advances enabling more accurate determinations of local gravitational acceleration, the development of refined computational models, and satellite gravimetry [16], standard gravity (*g*) continues to be widely used in many measurement devices and applications. However, the acceleration of gravity (*g*) is not a constant that remains invariable on Earth, so the result obtained may be different for the same applied force depending on the test location [17].

The study of the Earth’s gravity field models remains highly relevant today in the context of the rapid advancement of technologies and research techniques aimed at addressing scientific and practical challenges across diverse fields of knowledge—including environmental sciences and sports science. Recent progress in high-precision gravimetric measurements, both terrestrial and satellite-based, has enabled the development of detailed representations of the Earth’s gravity field at various spatial scales and resolutions tailored to the specific requirements of each investigation [17,18]. The continuous evolution of geodesy, along with advancements in both ground-based and spaceborne instrumentation, now allows for the integration of realistic gravity field data into any scientific analysis where gravity is a relevant variable. This approach is crucial for achieving greater accuracy and robustness compared to analyses that rely on constant or simplified gravity values.

The value of gravity varies around the Earth, from one location to another, depending on altitude, latitude, and other parameters such as the distribution of masses in the Earth’s crust in that area [18]. This variation in the gravity value is of a maximum of approximately 52 m·s^−2^ for a maximum latitude variation from the Equator to the Poles and of an approximate value of 0.3086·10^−5^ mGal per meter of variation in altitude (1 mGal = 10^−5^ m·s^−2^). Thus, on Earth, these gravity values, calculated as a function of the location’s latitude and altitude, fluctuate within a range of 9.7644 m·s^−2^ (Everest) and 9.8321 m·s^−2^ (Poles) (Supplementary Materials File S1).

To obtain a true gravity value at a given location, measurements must be taken with instruments called gravimeters and a reduction and correction procedure followed that is not trivial (Supplementary Materials File S1). However, it is also possible to obtain theoretical values taking into account a first-order approximation of the figure of the Earth. Successive terrestrial models have been presented for this purpose, defined by a rotation ellipsoid, the surface of which is the surface level of its own gravity field. This approach constitutes what is known as the Geodetic Reference System, adopted by the International Union of Geodesy and Geophysics (IUGG). The gravity field of this reference ellipsoid is uniquely defined for each location (via the latitude, longitude and height) and can be used as a good theoretical approximation of the true gravity value [17] (Supplementary Materials File S1). To this end, the IUGG currently recommends the Geodetic Reference System called GRS80 [17], widely used in geodetic studies and included in reference systems such as the European Terrestrial Reference System (ETRS89).

We must quantify the level of uncertainty in these measurements in order to assess whether a more accurate gravity value, calculated via the GRS80 system, determines significant differences in kinematic measurements in sports. This uncertainty depends not only on gravity but also on the time associated with the performance. The uncertainty, from the mathematical point of view, quantifies the inherent error of the result of a measurement, i.e., it informs us of the goodness of the values observed [19], which can differ significantly from the true values. Moreover, the uncertainty calculated for the estimation of a variable also quantifies the level of significance that could be relevant to the variation in its estimation. In this case of the power calculation, the variation is obtained when considering one gravity value and another (Supplementary Materials File S2).

Thus, based on the existing background regarding the variability of gravitational acceleration as a function of altitude and latitude, it is hypothesized that using the gravity value from the Geodetic Reference System known as GRS80, as opposed to the standard gravity value, improves the accuracy of jump power calculation.

The objectives of the present study were as follows:

- To evaluate the uncertainty in the jump power calculation induced by the direct measurements involved in its calculation.
- To calculate the power developed during the execution of three jump tests RJ (*P*_ow15_, *P*_ow30_, and *P*_ow60_) in the city of Cádiz (Spain) using a photoelectric cell system, applying the standard gravitational acceleration (*g* = 9.81 m·s^−2^).
- To calculate the required power output in the Reactive Jump (RJ) tests, this time applying the GRS80 Geodetic Reference System to data from 34 locations worldwide.
- To perform a comparison between the results obtained using the standard gravitational acceleration (*g* = 9.81 m·s^−2^) and those obtained using the GRS80 specific values of gravitational acceleration.

## 2. Materials and Methods

### 2.1. Participants

Ten male university volleyball players aged 18 years and over from the University of Cádiz (Spain) participated voluntarily in this study (age: 20.7 ± 1.51 years, height: 176.3 ± 19.0 cm, and weight: 65.4 ± 7.5 kg). Volleyball players were selected for the present study, given that the specific demands of this sport involve frequent training with repeated jumps of varying duration and intensity. This choice minimizes the risk of excessive fatigue during test execution. All were physically active, carrying out more than three exercise sessions per week at an amateur level. All subjects were fully informed about the study protocol, and written informed consent was obtained from all of them before the study in accordance with current national and international laws and regulations governing the use of human subjects in research (Declaration of Helsinki II). This study was approved by the University of Cádiz Doctoral Commission.

### 2.2. Procedure: Calculation of Uncertainty

We first describe the parameters involved in the calculation of jump power uncertainty. The measuring device used was an OptoJump infrared bars (Microgate Corporation, Bolzano, Italy) consisting of a transmitting optical bar and a receiver bar with a sensitivity of 10^−3^ s [11]. The use of this instrument is associated with an uncertainty or possible error in jumping power measurement induced by the direct measurements involved in its calculation, namely, flight time (tv), ground contact time (*tc*), and the local gravity value (*g*), as observed in the expression in [13]. The sensitivity of the OptoJump (Microgate Corporation, Bolzano, Italy) device for the measurement of flight and ground times is 10^−3^ s, and as regards the calculation of the local gravity value (GRS80), the sensitivity is 10^−8^ m·s^−2^. These values allow the error to be evaluated according to the procedure described in the international Guide to the Expression of Uncertainty in Measurement (GUM), which presents a homogeneous, rigorous, and unified treatment for its calculation [19]. Based on the GUM, the deviation of the measured value with respect to the true value was estimated by finding a linear approximation of the power expression and studying the propagation of error (Supplementary Materials File S2).

### 2.3. Tests of Measurement of Jump Power

The participants performed the jump tests in the sports hall of the University of Cádiz (Spain) on two different days with a 24 h interval. During the first session, the subjects carried out a familiarization session where they received instructions according to the protocol defined by Bosco et al. [13]. This session consisted of a 15 min warm-up that included gentle running, vertical jumping, and stretching. Finally, the subjects executed three repeated jump tests of 15, 30, and 60 s duration within the familiarization activities. A rigorous protocol must be followed for these tests, where the subject has to execute jumps at maximum intensity and height during the predetermined time (i.e., 15, 30, 60 s) without wasting time between jumps and while maintaining a flexion angle in the knee of 90° for each jump. In addition, hands must be kept resting on hips throughout the test and the jump area entered after a previous jump.

In the second session, the participants performed the three jump tests using the OptoJump infrared bar device (Microgate Corporation, Bolzano, Italy). The order of the three tests (15, 30, and 60 s) was randomized for each participant to minimize the potential effects of fatigue on performance. There was a five-minute break between the completion of each test to avoid excessive muscle fatigue.

**Figure 1:**
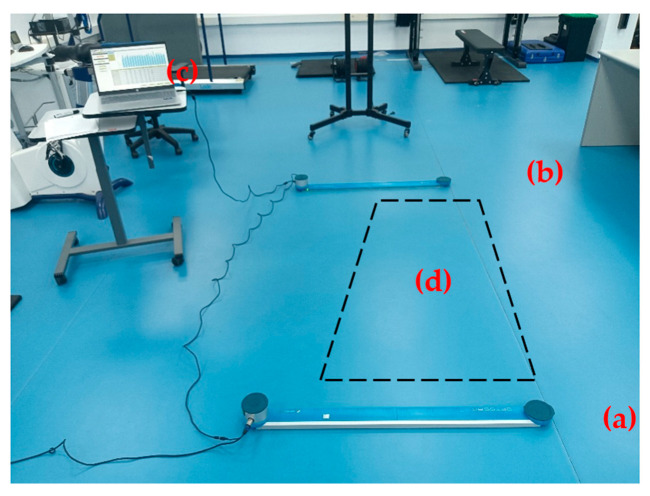
Opto-Jump device setup. The image shows the LED barriers, emitter (**a**), and receiver (**b**), separated by a distance of 3 m and connected to a laptop (**c**). Jump test execution area (**d**).

The OptoJump photoelectric system consists of a transmitting bar and a receiving bar, each equipped with 96 infrared LEDs (wavelength: 890 nm) positioned 3 mm above ground level and spaced at 1.05 cm intervals. These sensors detect flight time (*tv*) and ground contact time (*tc*) for each jump (Figure 1). The barriers were placed 3 m apart, allowing the athlete to perform the jumps comfortably without the need to worry about stepping outside the jumping area during the test execution. Each bar measures 100 × 4 × 3 cm and weighs approximately 2 kg. The OptoJump bars were connected to a personal computer, and jump height, contact time, and flight time were quantified using Microgate software (OptoJump software, version 1.13.4/2023). The system recorded *tv* during vertical jumps at a sampling rate of 1000 Hz. This calculation assumes that the center of mass is at the same position at both take-off and landing [20].

There was a five-minute break between the completion of each test to avoid excessive muscle fatigue. Previously, the subjects performed a 15 min warm-up consisting of continuous running, multi-jumping, and stretching. Data were obtained from each of the ten athletes for each test for the following different variables: flight time (*t_v_*), contact time (*t_c_*), number of jumps performed (*n*), and total test time (*t_t_*).

Two options were considered to calculate the power and performance developed (*P*_ow15_, *P*_ow30_, and *P*_ow60_) for these different tests. On the one hand, the power for each test was measured via a kinematic system considering the standard gravity value *g* = 9.81 m·s^2^.

On the other hand, the theoretical acceleration of local gravity *g* for this location, calculated according to the GRS80 model, was used to perform the power calculation taking into account the location of the athletes in Cádiz (Spain). Considering the latitude value of *φ* = 36°.4617 N and altitude *H* = 4.0 m and using the formulas corresponding to the GRS80 gravity, a value of *g* = 9.79857796 m·s^−2^ was obtained (Supplementary Materials File S1).

With this local gravity value, considering the geographic location of the experiment, the power developed by all subjects in each jump test (*P*_ow15_, *P*_ow30_, and *P*_ow60_) was calculated from Equation (2), which was applied to the data of each test. Table 1 shows the variables collected during the jump tests presented as mean ± standard deviation and the average of the calculated power values for the ten athletes in each test, considering the standard gravity value and using the local gravity value.

### 2.4. Measurement of Jump Power According to Different Geographic Locations

To analyze the influence of geographic location on power calculation, 34 additional global locations (Table 2) were subsequently selected to simulate the same jump tests conducted in Cádiz (Spain) but using the local gravitational acceleration of each site. The objective was to estimate the power that athletes would need to produce at these locations in order to achieve the same performance.

These 34 locations were chosen based on their relevance, including cities that have previously hosted the Olympic Games or World Athletics Championships, as well as locations that exhibit extreme gravitational values due to their geographic position on the globe.

If standard gravity is used in the power calculation, the resulting values would be identical across all cities, showing no variation due to the athlete’s location.

The following formula was applied to compute the average power difference for each jump test and for each location *i* (*i* = 1…34) relative to Cádiz using the local gravitational acceleration values:

*P^i^_diff_* = (*g_i_*^2^ − *g_c_*^2^) · *k     i =* 1,…,34 (3)

where *k* is

*k* = *n* · (*t_v_* · (*t_v_* + *t_c_*))/(4 · *t_c_*)

*g_i_* and *g_c_*: Local gravity value in the i-location and in Cádiz, respectively.

*n*: Average number of jumps performed in the test.

*t_v_:* Average flight time obtained in the test.

*t_c_*: The average contact time obtained in the test.

### 2.5. Statistical Analysis: Measurement of Jump Power According to Different Geographic Locations

Descriptive statistics were calculated for all variables and expressed as mean ± standard deviation. Prior to inferential analyses, data distribution normality was assessed using the Shapiro–Wilk test for each test group (*P_ow15_*, *P_ow3_*_0_, and *P_ow60_*). Homogeneity of variance was evaluated using Levene’s test to verify ANOVA assumptions. Differences among the three jump tests were analyzed using a one-way ANOVA with test type as the independent variable (three levels: *P_ow15_*, *P_ow30_*, and *P_ow60_*) and total power as the dependent variable. When significant main effects were detected, the Bonferroni post hoc test was conducted for pairwise comparisons to identify specific differences between test durations.

The statistical significance of power differences between standard gravity (*g* = 9.81 m·s^−2^) and local gravity values was determined using the measurement uncertainty framework established in Supplementary Materials File S2. Power differences were compared against the coverage error (±2 standard deviations) derived from the uncertainty analysis for each location and test duration. A difference was considered statistically significant when the absolute power difference exceeded twice the coverage error for the specific test (*P_ow15_*: 1.24 W, *P_ow30_*: 1.78 W, *P_ow60_*: 2.28 W). Z-scores were calculated as the ratio of power difference to coverage error, and *p*-values were derived using the standard normal distribution.

The cumulative error was calculated as the sum of individual power differences between the standard gravity value (*g* = 9.81 m·s^−2^) and a range of local gravity values (9.764 to 9.832 m·s^−2^, incremented by 0.001 m·s^−2^) across all jumps performed in each test. For each gravity value in the range, the individual error was computed as the difference between power calculated using standard gravity and power calculated using the specific local gravity value. The cumulative error represents the total accumulated difference across the entire range of gravity values, calculated using the following procedure (Formulas (4)–(7)):

Calculate reference power using standard gravity: *P_ref_* = (*g^2^·t_v_·t_t_*)/(4·*t_c_*) (4)

For each local gravity value (*g*_i_), calculate P_i_ = (*g_i_^2^·t_v_·t_t_*)/(4·*t_c_*) (5)

Compute individual error: ε_i_ = *P_ref_ − P_i_* (6)

Sum all individual errors: cumulative error = Σεi(7)

where *t_v_* is flight time, *t_c_* is contact time, and *t_t_* is total time (*t_v_* + *t_c_*).

Percentage error was calculated using Formula (8):

Percentage error = ((power_city − power_g)/power_g) × 100(8)

where power city represents estimated power at a specific location and power *g* represents power under standard gravity conditions.

Individual response variability was assessed by calculating power differences for each participant across different gravity conditions (Everest, standard, and Poles) relative to Cádiz values. Inter-individual variability was quantified using coefficients of variation (CVs), calculated as (standard deviation/mean) × 100 for power differences within each test and gravity condition. Individual consistency was evaluated by calculating the standard deviation of power differences across gravity conditions for each athlete, where lower values indicate more consistent responses. Correlations between gravity conditions were computed using Pearson correlation coefficients to assess the proportional consistency of individual responses to gravitational variation.

A significance level of α = 0.05 was set for all hypothesis testing. All the analysis was conducted using the R programming language for statistical computing (version 4.2.2).

## 3. Results

Statistically significant differences (*p* < 0.001) were found in the variables number of jumps (*n*), flight time (*t_v_*), contact time (*t_c_*), total test time (*t_t_*), and total power developed between the three jump tests (*P*_ow15_, *P*_ow30_, and *P*_ow60_). A statistically significant difference (*p* > 0.05) was also found in the variable jump contact time (*t_c_*) (Table 1).

Statistical analysis revealed significant differences between standard and local gravity calculations in 22, 25, and 28 cities for *P_ow15_*, *P_ow30_*, and *P_ow60_* tests, respectively, with significance levels increasing with test duration (Table 2). The difference in value with respect to the power calculated in the city of Cádiz (Spain), where the test was carried out, is denoted in parentheses.

The results show that the differences in power calculated with respect to Cádiz (Spain), where the tests were performed, ranged between −4.55 W and 5.09 W for the *P*_ow15_ test, between −8.14 W and 10.70 W for *P*_ow30_, and between −14.65 W and 17.90 W for *P*_ow60_ (Table 2).

**Figure 2:**
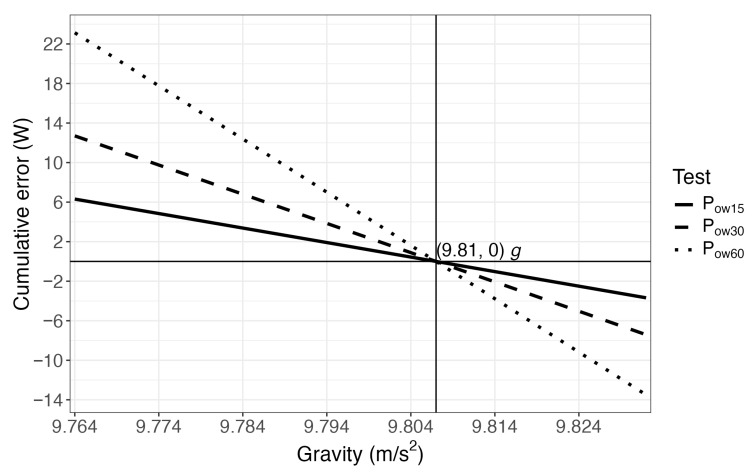
Cumulative error comparison by gravity calculation method.

Figure 2 shows the accumulated error in the calculation of the power for each test (*P_ow15_, P_ow30_,* and *P_ow6_*_0_) when using the local gravity value versus standard gravity value (*g* = 9.81 m·s^−2^).

**Figure 3:**
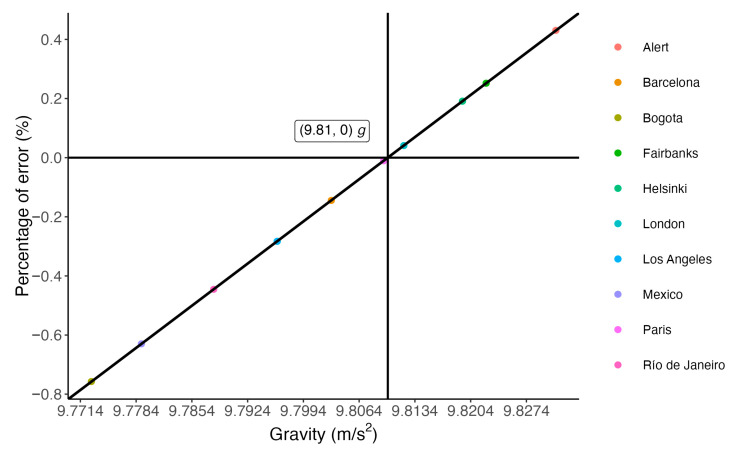
Percentage error relative to standard gravity.

Figure 3 shows the percentage error (%) with respect to the standard value of gravitational acceleration, *g* = 9.81 m·s^−2^ for the following locations: Alert (Canada), Barcelona (Spain), Bogotá (Colombia), Fairbanks (USA), Helsinki (Finland), London (UK), Los Angeles (USA), Mexico City (Mexico), Paris (France), and Rio de Janeiro (Brazil). The more the value of local gravity deviates from the standard value, the greater the error it causes. The percentage of error is defined according to Formula (8).

**Figure 4:**
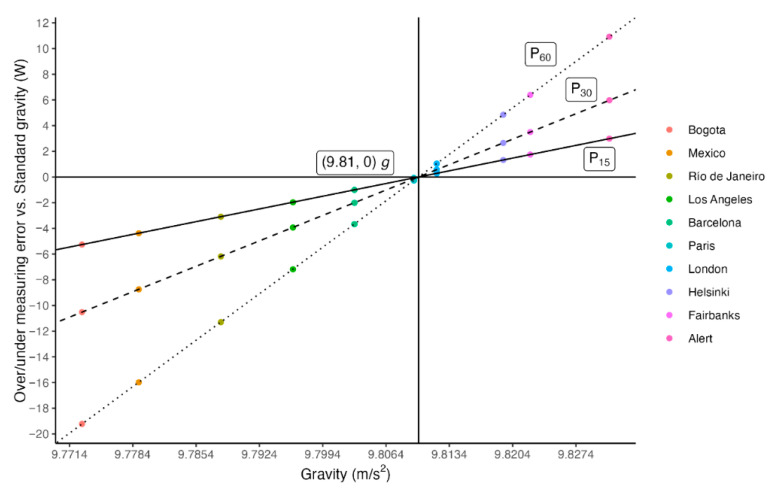
Power value comparison across locations.

The differences between power values calculated using the local gravity value and in different locations for each test (*P*_ow15_, *P*_ow30_, and *P*_ow60_) are shown in Figure 4.

Figure 4 shows the comparison of power values calculated using the standard value of *g* = 9.81 m·s^−2^ and the over/underestimation of the required power in other cities to obtain the same result, taking into account local gravity values according to GRS80.

Individual analysis across the 10 participants revealed uniform directional responses to gravitational variation, with perfect correlations (r = 1.0) between gravity conditions for all tests. Inter-individual variability was moderate (CV = 21.9–25.5%), with individual consistency scores ranging from 7.56 to 13.7 W. All athletes demonstrated the expected pattern of decreased power at high-altitude locations and increased power at polar locations, confirming that gravitational effects are consistent across individuals despite magnitude differences.

## 4. Discussion

The objectives of this study were, first, to evaluate the uncertainty in the jump power calculation induced by the direct measurements involved and, second, to calculate the power developed during the performance of three Reactive Jump (RJ) tests (*P_ow15_*, *P_ow30_*, and *P_ow60_*) in the city of Cádiz (Spain) using LED–sensor barriers and the standard gravitational acceleration value (*g*) of 9.81 m·s^−2^. Subsequently, the power required for the same performance and protocol was calculated for 34 other locations on Earth, taking into account the local *g* values according to the GRS80 system. Finally, these results were compared with the power values obtained using the standard gravity value of *g* = 9.81 m·s^−2^.

Power measurement systems, such as opto-electric bars and force platforms, have proven to be precise in quantifying flight time, take-off speed, and applied force; however, some studies have reported measurement errors of up to 0.022 m in jump height using different methods and devices [20,21,22,23,24].

Several studies have evaluated the validity and reliability of systems based on infrared barriers, such as OptoJump^TM^, in comparison to force platforms [25]. These studies have reported high correlation values, with only minor deviations in measured contact and flight time. Other research suggests the use of predictive equations, allowing coaches and researchers to employ OptoJump for assessing vertical jump performance using linear regression models tailored to each specific jump modality [20].

In addition to these predictive equations and linear regression models, we consider that using the local gravitational acceleration value instead of the standard gravity (*g*) should similarly be recommended in order to reduce calculation error and improve measurement accuracy.

It is well known that the use of power measurement systems is associated with an uncertainty or possible error in jump power measurement induced by the direct measurements involved in their calculation, such as flight time (*t_v_*), ground contact time (*t_c_*), and the gravity value (*g*). However, the uncertainty in the power calculation from the random error of the measurements is small compared with the differences in the power values obtained considering different gravity values according to the location of the athlete (Supplementary Materials File S2). Consequently, all the differences described in this study were significant and cannot be associated with the act of measuring.

In our study, the results demonstrate notable differences in the calculated power when using the standard gravitational acceleration value (*g* = 9.81 m·s^−2^) compared to the location-specific gravitational values derived from the GRS80 model. Specifically, when comparing data computed for the city of Cádiz (Spain) with other locations, significant variations were observed: from −4.55 W to 5.09 W in the *P_ow15_* test, from −8.14 W to 10.70 W in *P_ow30_*, and from −14.65 W to 17.9 W in *P_ow60_* (Table 2).

Moreover, discrepancies in power values calculated using standard gravity versus local gravity (GRS80 model) ranged from −5.26 W to 2.99 W for *P_ow15_*, −10.52 W to 5.98 W for *P_ow30_*, and −19.22 W to 10.93 W for *P_ow60_* (Figure 3). These findings highlight the influence of geographic gravitational variation on power calculations, emphasizing the relevance of using accurate local gravitational values instead of the standard *g* in biomechanical assessments of jump performance.

It is observed that different power values are obtained for the same flight time, ground and number of jumps when using real gravity instead of standard gravity. That is to say, if we use the standard gravity value, it is evident that with the same flight time, ground and contacts, the same power would be obtained, but if we use the real gravity value, the power developed would be different depending on the test location. Thus, we can see that the greater the difference between the standard gravity value and the local real gravity value, the greater the error, and this increases as the duration of the test increases. Therefore, the longer the test lasts, the greater the overestimation or underestimation of the power developed by the athlete.

Importantly, the gravity value used for calculating jump height for jump capacity measurement systems such as the infrared bars used in this study, as well as many other instruments, will in most cases be predetermined and defined in the software employed. In this way, these instruments consider the acceleration of gravity as constant, regardless of where the jump test is performed, with a value of 9.81 m·s^−2^ [13].

If we focus on the variable of gravity value *g*, we must take into account that the lowest value occurs at Mount Everest, with a theoretical GRS80 gravity *g* = 9.76444176 m·s^−2^. Currently, power measurements are not being carried out at this location; however, there are similar locations such as Bogotá (Colombia) with more than 7 million inhabitants and a value of *g* = 9.7727797 m·s^−2^ and Olympic city of Mexico City with *g* = 9.77906297 m·s^−2^ where sports competitions are commonly held. If we compare the power values obtained in Cádiz (Spain) with those required in the city of Bogotá, we find that we would require −3.64 W, −7.28 W, and −13.31 W for the *P*_ow15_, *P*_ow30_, and *P*_ow60_ tests, respectively (Table 2).

On the other hand, the highest *g* value occurs at the Poles, *g* = 9.83218637 m·s^−2^, with the nearest habitable location being Alert (Canada), *g* = 9.83109609 m·s^−2^, which is the northernmost permanently inhabited human settlement on Earth. In turn, the nearest Olympic city (1952) is Helsinki (Finland), which was also the venue of the World Athletics Championships (2005), with a value of *g* = 9.81936464 m·s^−2^. At this location, higher power values would have been required for the execution of the same tests: 2.94 W, 5.89 W, and 10.76 W, respectively (Table 2).

The differences found need not be compared across different countries or continents as important differences can be found in the local gravity values within each country. For example, within the United States, *g* values can vary greatly (Los Angeles with *g* = 9.79610872 m·s^−2^ and Fairbanks *g* = 9.82234596 m·s^−2^).

If we analyze the differences in the power values calculated with local *g* values (*P*_ow15_, *P*_ow30_, and *P*_ow60_) and those calculated using the value *g* = 9.81 m·s^−2^, we find two situations. First, for the city of Bogotá (Colombia), where *g* = 9.77277970 m·s^−2^, the difference in power used is lower, with values of −5.35 W, −10.67 W, and −18.58 W, respectively. Second, comparing this location with a site with a higher *g* value, Alert (Canada) *g* = 9.831096609 m·s^−2^, greater power values are required for the same jump performance: 2.9 W, 5.82 W, and 11.57 W. In addition, we must consider that the tests lasted just 15, 30, and 60 s, and even greater differences would be estimated for longer test durations, as reported in other works [6,21]. Our results also coincided with those of other studies in which mathematical simulations were performed, reproducing the effects of altitude on the 100 and 200 m speed tests. This research demonstrated that the altitude effect produced for the 200 m tests was significantly greater than for 100 m, suggesting that the duration of the test was also a determinant of the result and that perhaps the legality of such records should be reconsidered [23]. Methodological and instrumental errors should not be subject to measurement errors greater than the differences that the researcher aims to find between various individuals, or in the same subject, to establish possible improvements, however small they may be [21]. Thus, we also consider it necessary to tend to the smallest difference, as occurs in sports competitions with a photo finish to determine performance (sensitivity of 0.0005 s), by using more accurate gravity values to improve the accuracy of the measurements conducted in different kinematic and kinetic studies.

The standard gravity value has been used for several decades in research measuring jumping ability [14], but it is also used today in instruments that assess jumping ability using photocell devices, where other measurement errors have also been observed [20]. Currently, mobile phone applications such as the app “My Jump” use the standard value of gravity (*g* = 9.81 m·s^−2^) for their calculations [26]. Therefore, depending on the location where the app is used, there may be some measurement error in the calculation of power for the same jump height. To this end, we recommend the computer programs of the different devices employed for quantifying sports performance, whether jump bars, dynamometric platforms, or other similar systems, incorporate a menu that permits the incorporation of local gravity values or an electronic device capable of quantifying the gravity value as a function of the site’s altitude using the local gravity value with the GRS80. In practical terms for coaches and researchers, it is possible to obtain more accurate estimates of jump power by creating spreadsheets that incorporate Formula (2), the flight and contact time of the jump, and the local gravitational acceleration specific to the location where the assessment is conducted.

Similarly, the various devices used to calculate power or other physical parameters should incorporate into their software the formula for computing gravity based on the athlete’s location according to the GRS80 model. To do this, it is only necessary to know the latitude, longitude, and altitude coordinates of the athlete’s location—values that are easily accessible—and apply the formulas provided in Supplementary Materials File S1.

Accordingly, if the altitude at which the competition takes place or wind speed are taken into account to validate a sports performance, then a laboratory setting is even more crucial for implementation. In such an environment, the different measurement systems quantify sports performance through kinematic and dynamic values, and the altitude at which the measuring device is located can affect the result. Thus, there is a significant need to include more accurate gravity values in the formulas for calculating both jump capacity and power. Without this consideration, a subject’s performance may vary for the same applied force depending on the test location, causing the accumulated systematic error produced to be accentuated as the duration of the physical test increases.

Thus, in response to the proposed hypothesis, the differences observed in jump power calculations when using the standard gravity value versus the GRS80 model suggest that the latter should be adopted to improve measurement accuracy. It is important to emphasize that the longer the test duration and the greater the difference between the local gravity value and the standard reference, the larger the accumulated error and the discrepancy in power output (Table 2; Figure 2, Figure 3 and Figure 4).

The main strength of the study lies in being the first to calculate the actual power output developed by an athlete following vertical jump tests, taking into account the local gravitational acceleration according to GRS80, and compare it with the error produced by using the standard gravity value. The results obtained indicated that the geographic location of the experiment should be considered and the GRS80 local gravity values should be used to improve the accuracy of jump power calculation.

This study’s main limitation is the impossibility of reproducing, under real conditions, one athlete’s performance at the different locations for which the local gravity value was calculated. Therefore, the calculation in this study was used to simulate the power required to perform the same jump test in different locations with different local *g* values.

Future research should focus on evaluating the use of the gravitational value in other devices or systems that assess athletes’ physical condition. As a future line of research, it would be advisable to conduct multi-center studies involving sports centers located at different altitudes. Such an approach would allow athletes to perform the same test under varying gravitational conditions, enabling a more comprehensive evaluation of how local gravity influences the assessment of power output. This would further validate the relevance of incorporating site-specific gravitational values into biomechanical analyses of jump performance.

## 5. Conclusions

Power is a critical factor in sports performance and is commonly calculated using the gravitational value of *g* = 9.81 m⋅s^−2^. However, this “*g*” value varies with altitude and geographical latitude. The use of this average gravity value results in a loss of accuracy in jump power measurements.

Given the difficulty of obtaining an actual gravity value at each specific test location, this study demonstrates that the Geodetic Reference System GRS80—endorsed by the International Association of Geodesy (IAG)—enables the calculation of a theoretical gravity value based on the geographic coordinates (latitude and longitude) and the altitude of the site. This calculated value serves as a more accurate substitute for the standard constant gravity approximation traditionally used in the calculation of an athlete’s jump power.

With these considerations, this study demonstrated an improvement in the accuracy of the jump power calculation using the GRS80 local gravity value. The differences observed indicated that GRS80 local gravity values should be used to improve the accuracy of jump power calculation.

It is recommended that devices currently using a standard gravitational value (g = 9.81 m⋅s^−2^) in their software replace it with the local gravity value based on the GRS80 model. This adjustment will improve the accuracy of sports performance assessments.

## Figures and Tables

**Table 1:** Descriptive analysis of jump power tests in Cádiz (Spain); *g* = 9.79857796 m·s^−2^.

Variable	*P*_ow15_	*P*_ow30_	*P*_ow60_
Number of jumps (*n*)	16.9 ± 1.4	35.4 ± 3.2	74.9 ± 6.3
Total flight time (s)	9.7 ± 0.8	19.6 ± 1.7	38.1 ± 3.2
Jump flight time (s)	0.57 ± 0.04	0.55 ± 0.05	0.51 ± 0.05
Contact time (s)	4.9 ± 0.6	10.0 ± 1.5	21.6 ± 3.2
Jump contact time (s)	0.29 ± 0.07	0.28 ± 0.07	0.29 ± 0.06
Total power (W)	714.1 ± 168.4	1439.4 ± 367.3	2613.1 ± 572.9

The results are expressed as mean ± standard deviation. The variables flight/jump contact time indicate the average flight/contact time per test. *p* < 0.001 for all variables except jump contact time (s) (*p* > 0.05).

**Table 2:** Local gravity values and power calculations with statistical significance compared to standard gravity (*g* = 9.81 m·s^−2^). The locations with the values of minimum power (Everest), maximum (Poles), standard gravity value g, and place of performance of the tests (Cádiz, Spain) are also shown in bold.

Cities/Places	*g* Value (m·m^−2^)	*P_OW15_* Value [Difference] (W)	*P_OW30_* Value [Difference] (W)	*P_OW60_* Value [Difference] (W)
**Everest (India)**	9.76444176	687.75 [−4.55] ***	1377.09 [−8.14] ***	2517.91 [−14.65] ***
Bogotá (Colombia)	9.77277970	689.00 [−3.64] ***	1378.16 [−7.28] ***	2518.80 [−13.31] ***
Eldoret (Kenya)	9.77384331	689.15 [−3.49] ***	1378.46 [−6.98] ***	2519.35 [−12.76] ***
Mexico City (Mexico)	9.77906297	689.89 [−2.75] ***	1379.94 [−5.51] ***	2522.04 [−10.07] ***
Quito (Ecuador)	9.78031807	690.07 [−2.58] ***	1380.29 [−5.16] ***	2522.69 [−9.42] ***
Rio Janeiro (Brazil)	9.78813727	691.17 [−1.47] ***	1382.50 [−2.95] ***	2526.72 [−5.39] ***
Miami (USA)	9.78987641	691.42 [−1.23] ***	1382.99 [−2.46] ***	2527.62 [−4.49] ***
Brisbane (Australia)	9.79122651	691.61 [−1.04] ***	1383.37 [−2.08] ***	2528.32 [−3.79] ***
Atlanta (Georgia)	9.79522080	692.17 [−0.47] ***	1384.50 [−0.95] ***	2530.38 [−1.73] ***
Los Angeles (USA)	9.79610872	692.30 [−0.35] **	1384.75 [−0.70] ***	2530.84 [−1.27] ***
Sydney (Australia)	9.79642474	692.34 [−0.30] **	1384.84 [−0.61] ***	2531.00 [−1.11] ***
Tokyo (Japan)	9.79792548	692.55 [−0.09] **	1385.26 [−0.18] ***	2531.78 [−0.33] ***
**Cádiz (Spain)**	9.79857796	692.65 [0.00] **	1385.45 [0.00] ***	2532.11 [0.00] ***
Athens (Greece)	9.79897530	692.70 [0.06] *	1385.56 [0.11] ***	2532.32 [0.21] ***
Seoul (S. Korea)	9.79927909	692.75 [0.10] *	1385.65 [0.20] ***	2532.48 [0.37] ***
Melbourne (Australia)	9.79953785	692.78 [0.14] *	1385.72 [0.27] ***	2532.61 [0.50] ***
Madrid (Spain)	9.80003366	692.85 [0.21] *	1385.86 [0.41] **	2532.87 [0.76] ***
St. Louis (USA)	9.80004259	692.85 [0.21] *	1385.86 [0.42] **	2532.87 [0.76] ***
Beijing (China)	9.80141854	693.05 [0.40]	1386.25 [0.81]**	2533.58 [1.47] ***
Barcelona (Spain)	9.80290510	693.26 [0.61]	1386.67 [1.23] *	2534.35 [2.24] **
Rome (Italy)	9.80332055	693.32 [0.67]	1386.79 [1.34] *	2534.57 [2.46] **
Montreal (Canada)	9.80656346	693.78 [1.13]	1387.71 [2.26]	2536.24 [4.13]
Munich (Germany)	9.80759672	693.92 [1.28]	1388.00 [2.55]	2536.78 [4.67]
Paris (France)	9.80947844	694.19 [1.54]	1388.53 [3.09]	2537.75 [5.64]
**Standard g**	9.81	694.37 [1.71]	1388.81 [3.39]	2537.41 [5.27]
Antwerp (Belgium)	9.81174842	694.51 [1.86]	1389.18 [3.73]	2538.93 [6.82]
London (England)	9.81201004	694.55 [1.90]	1389.25 [3.80]	2539.06 [6.95]
Berlin (Germany)	9.81269041	694.64 [2.00]	1389.44 [4.00]	2539.41 [7.30]
Amsterdam	9.81275173	694.65 [2.01]	1389.46 [4.01]	2539.45 [7.34]
Moscow (Russia)	9.81522041	695.00 [2.36]	1390.16 [4.71]	2540.72 [8.61] *
Stockholm (Sweden)	9.81857397	695.48 [2.83]	1391.11 [5.66] **	2542.46 [10.35] ***
Helsinki (Finland)	9.81936464	695.59 [2.94] *	1391.33 [5.89] **	2542.87 [10.76] ***
Fairbanks (USA)	9.82234596	696.01 [3.37] **	1392.18 [6.73] **	2544.41 [12.30] ***
Alert (Canada)	9.83109609	697.25 [4.61] ***	1394.66 [9.21] ***	2548.95 [16.84] ***
**Poles**	9.83218637	698.39 [5.09] ***	1395.75 [10.70] ***	2550.63 [17.90] ***

Note: Differences compared to standard gravity (*g* = 9.81 m·s^−2^). Statistical significance: *** *p* < 0.001, ** *p* < 0.01, * *p* < 0.05.

## Data Availability

The authors report that the supplementary material contains the formulas and complementary procedures used in the present study, which form the basis for the results presented in the main manuscript. For further inquiries or additional information, please do not hesitate to contact the corresponding author.

## Supplementary Materials

The following supporting information can be downloaded at: https://www.mdpi.com/article/10.3390/s25165163/s1, Supplementary Materials File S1: Calculation of the gravity value, Supplementary Materials File S2: Calculation of uncertainty associated with power measurement. [27,28,29,30,31]
