# Supplementary material for: Enhanced Accuracy in Jump Power Estimation Using Photoelectric Cell System and GRS80 Location-Specific Gravitational Acceleration
Source: Sensors (Basel). 2025 Aug 20;25(16):5163. doi: 10.3390/s25165163 (PMC12389838; doi:10.3390/s25165163)
Supplement: Supplementary file 1 [file sensors-25-05163-s001.zip › Supplementary Materials File S1.pdf]

Supplementary Materials File S1:
Calculation of the gravity value

J.L. González-Montesinos [1], F.G. Montesinos[2,3], J.R. Fernández Santos[1], A. Suárez Llorens[4], I. Caraballo[1,*], P. Gutiérrez Mulas[5], J.V. Gutiérrez-Manzanedo[1].

[1]Department of Physical Education, Faculty of Education Sciences, University of Cádiz (11510 Cádiz, Spain).
[2] Faculty of Mathematics Sciences, Complutense University of Madrid (28040 Madrid, Spain).
[3] Research Group "Geodesia", Complutense University of Madrid (28040 Madrid, Spain).
[4] Department of Statistics and Operation Research, University of Cádiz (11510 Cádiz, Spain).
[5] Faculty of Physical Activity and Sport Sciences. Complutense University of Madrid- (28040 Madrid, Spain).

* Corresponding author: Israel Caraballo Vidal. e-mails: israel.caraballo@uca.es

Calculation of the gravity value

The force of gravity *g* on or near the surface of the earth is derived from the forces exerted by the Earth, as a heterogeneous mass (*M)* in rotation, on any point on its surface or at a certain height (*h*).

It corresponds to the sum of the force of gravitational attraction ($\boldsymbol{F}_G$), defined by Newton's law of universal gravitation, and centrifugal force ($\boldsymbol{F}_C$), caused by the Earth's rotation, and its intensity is defined by:

$$g = F_G + F_C = G\frac{M}{d_p^2} + \frac{1}{2}\omega^2 \cos\varphi \quad (1)$$

G: Universal gravitation constant = 6.67259·10^{-11} *m kg*$^{-1}$ *s*$^{-2}$.

d_p*: Point position vector radius, defined according to altitude and latitude.*

M: Earth's mass.

ω: Earth's rotation speed.

φ: Latitude of the observation site.

Thus, the force of gravity is a vector, the direction of which is tangential to that known as the direction of the plumb line that orthogonally cuts the equipotential surfaces of the gravity field and the modulus of which corresponds to the acceleration of gravity. This vector depends both on the position, mainly the latitude and altitude of the point where it is determined, and on the distribution of terrestrial masses (atmosphere, hydrosphere, cryosphere, crust, mantle, and terrestrial core) and the position of extraterrestrial masses, mainly the Sun and Moon. In this way, the gravity value also suffers temporal variations due to possible changes that may occur in any of these parameters. While the position of the test site is easy to determine more or less immediately via geolocation systems, this is not the case for acquiring the gravity value in any location due to the distributions of terrestrial masses or the effect of extraterrestrial masses on the gravity value (mainly the so-called Earth or gravimetric tides); this knowledge requires a detailed and more complex study since the Earth is a heterogeneous and dynamic body.

The geoid is defined (Figure 1) from the Earth's gravity field and its corresponding potential as the level surface (equipotential surface of the gravity potential) that coincides with the mean level of the oceans, extending under the continents, on an idealized surface in hydrostatic equilibrium and in the absence of external forces [1]. In this way, the geoid, which represents the mathematical figure of the Earth, is a reflection of the variability in the gravity field at any point on Earth.

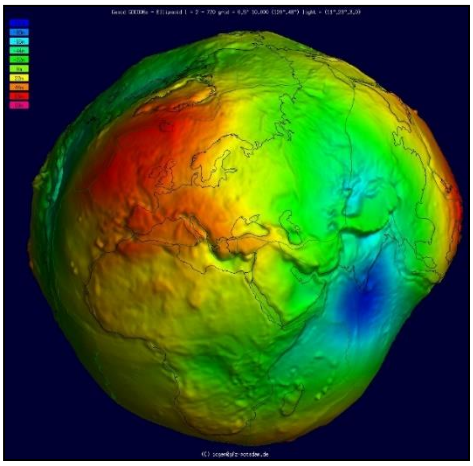

Figure 1 S1: Global Geoid Ripple Model GOCO06c, International Centre for Global Earth Models [2].

To obtain a true gravity value for a given location, measurements must be taken with gravimeters. Subsequently, these measured values are processed and corrected, eliminating the effect of Earth tides and ocean tide loading, among other effects and in accordance with the study planned in order to determine a true gravity value for the location in question that is independent of time.

However, it is also possible to obtain theoretical values taking into account a first-order approximation of the figure of the Earth. To study the figure of the Earth and its gravity field, a rotation ellipsoid of homogeneous mass, which approximates the level surface of the Earth gravity field, is used as a reference model. In this way, successive terrestrial models have been introduced and defined by a rotation ellipsoid, the surface of which is the level surface of its own gravity field, constituting what is known as the Geodetic Reference System. The gravity field of this reference ellipsoid, known as the "*normal gravity field*", is then uniquely defined for each point outside the ellipsoid and is easily calculated from four parameters that define the ellipsoid: total mass, angular velocity, and two geometric coefficients such as the semimajor axis and flattening.

Since the 1970s, the IUGG has recommended the Geodetic Reference System called GRS80 [3], widely used in geodetic works and included in reference systems such as the European (ETRS89). This ellipsoid is defined by a geocentric gravitational constant of the Earth, *GM*, with a value of $398600.5 \cdot 10^9$ $m^3 \cdot s^{-2}$, rotation speed of $7.292115 \cdot 10^{-5}$ rad/s, semimajor axis of $6.378137 \cdot 10^6$ m, and flattening of $2.98257722 \cdot 10^2$.

From this definition and Somigliana's formula [4], the value of normal gravity at any point on the surface of the ellipsoid GRS80 (ellipsoidal altitude h = 0) is determined with the expression

$$\gamma_0 = 9.7803267715\,(1 + 0.005302440112\,\mathrm{sen}^2\,\varphi - 0.0000058\,\mathrm{sen}^2\,2\varphi) \qquad (2)$$

φ*: Latitude of the observation site.*

If we consider this approximation, which only considers the gravity variation due to the change in latitude, the gravity variation range is obtained with a minimum value in the Terrestrial Equator of γ_0 = 9.7803267715 $m \cdot s^{-2}$ and maximum at the Poles, with a value of γ_0 = 9.8321863685 $m \cdot s^{-2}$. Figure 2 shows the normal gravity map, considering ellipsoid GRS80 as an approximation of the Earth for points located on the ellipsoid.

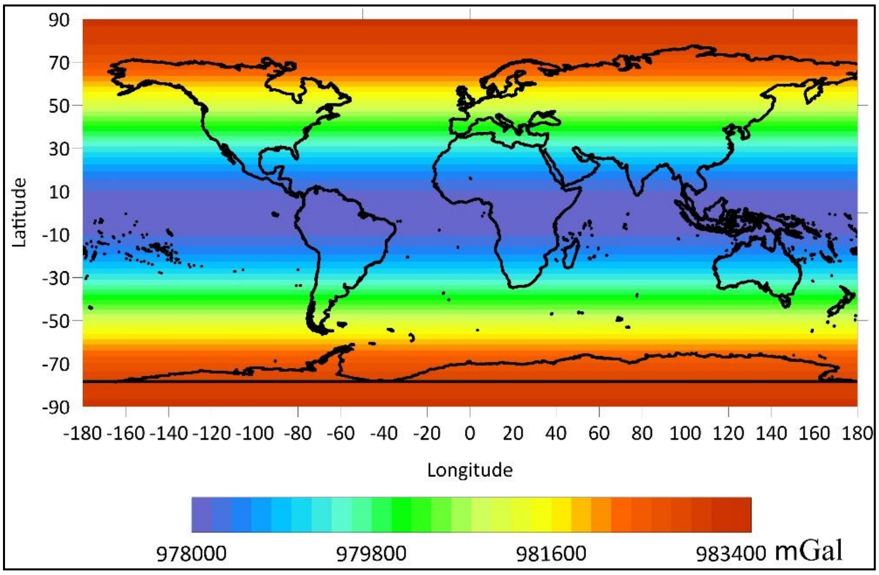

Figure 2 S2: Theoretical gravity values approximating the Earth to the GRS80 rotation ellipsoid. Calculations on the surface of the ellipsoid (zero altitude).

To achieve a better approximation of the true gravity value, we must also consider gravity variation with respect to the altitude of each location. In the case of the GRS80 ellipsoid, the reduction in gravity as a function of altitude is given by the expression (3a):

$$C_h = (0.308549 \times h + 0.0002198 \times \cos 2\varphi \times h - 0.721 \times 10^{-7} \times h^2) \times 10^{-5} \quad \text{m s}^{-2} \qquad (3a)$$

h: Altitude above the ellipsoid (m).

φ: Latitude of the observation site.

This value, C_h, must be subtracted from the corresponding normal gravity value (2) to obtain the normal gravity value for that given altitude and latitude, γ.

Although this formulation involves the ellipsoid altitude, *h*, of each location, we can consider the altitude above sea level, *H* (*orthometric altitude*), for the purposes of this work. The difference between ellipsoid and orthometric altitudes corresponds to the *undulation of the geoid*, the values of which can oscillate by a maximum of -100 to 100 m. This difference would imply a variation of 0.0002 m·s^{-2}, so the proposed approximation of considering orthometric altitudes can be assumed within the margin of precision required for the estimation of power in the present study.

Thus, the maximum variation of γ corresponding to differences in latitude (Equator-Pole) is 0.05186 m·s^{-2} and to elevation differences (sea level—Mt Everest at 8850 m) is 0.02725 m·s^{-2}.

Finally, to further approximate the calculated gravity value with reference to the true value, we must consider the gravitational attraction effect exerted by the mass of the atmosphere on the atmospheric station by virtue of the altitude, *H*, at which the station lies above sea level. This effect is of the opposite sign to that of the gravity exerted by terrestrial masses (in this case ellipsoid), so it must be incorporated into equation (4) with a minus sign, and its value, A_h, can be calculated via the following empirically determined formula (*NGA, 1999*):

$$A_H = 0.87\ e^{-0.116\, H^{1.047}} \quad \text{mGal (1 mGal} = 10^{-5}\ \text{m·s}^{-2}) \qquad (3b)$$

where H is the altitude of the station in km. This value can be, e.g., 0.87·10^{-5} m·s^{-2} or 0.77·10^{-5} m·s^{-2} for

H=1 km and $0.68 \cdot 10^{-5}$ $m \cdot s^{-2}$ for H=2 km. This is not a very significant value in this study; nevertheless, we incorporated it in order to obtain a gravity value as close as possible to the true value [5].

Figure 3 shows the gravity variation due to the effect of altitude (formulas 3a and 3b), assuming a fixed latitude position of 45º.

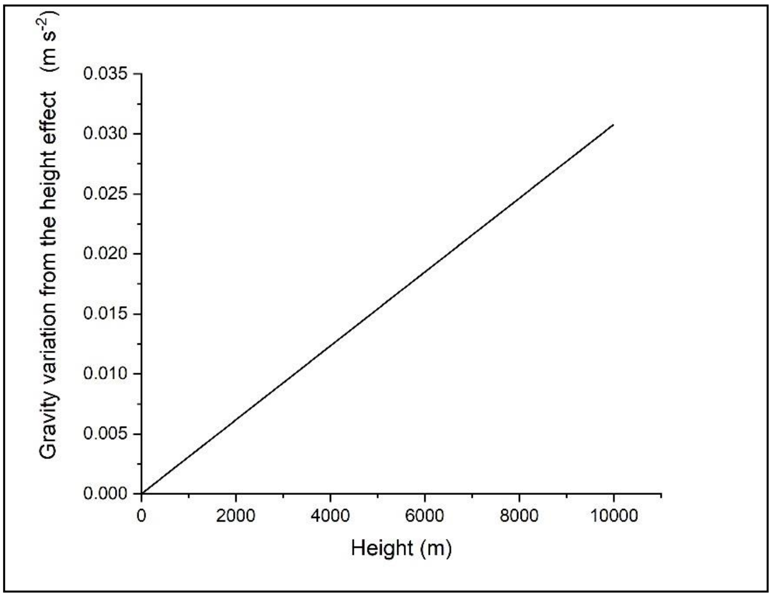

Figure 3 S1: Gravity variation in $m \cdot s^{-2}$ depending on the altitude of the station, assuming a fixed latitude of 45º, corresponding to the effects defined by C_H and A_H (formulas 3a and 3b).

The difference between the normal gravity value obtained for any location with coordinates (φ, λ, and H) and true gravity value at that same location is due to the difference between the Earth's true distribution of heterogeneous masses (mainly the most superficial area and that closest to the site in question) and the homogeneous mass of the ellipsoid considered the approximation. These differences can be of the order $5 \cdot 10^{-4} \cdot g$, so we can regard them as negligible in the present study and consider the calculation of the gravity effect from the ellipsoidal approximation as sufficiently precise for these studies.

The values of these gravity variations due to mass distribution are identified from the so-called gravity anomalies, calculated as the difference between observed gravity and normal gravity corresponding to a reference ellipsoid (Figure 4).

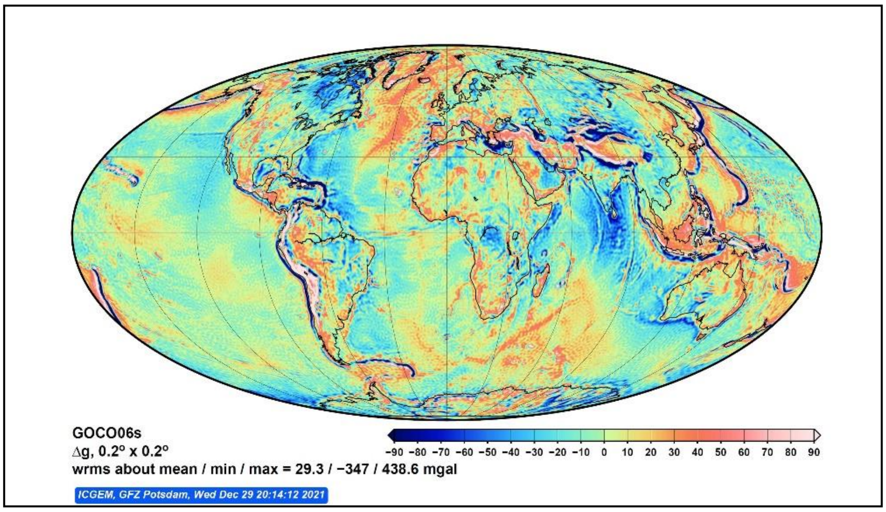

Figure 4 S1: Gravity anomalies considering the GOCO06c gravity model and the GRS80 ellipsoid.

On the other hand, periodic gravity variations due to the effect of the attraction of the Sun and Moon and that produced by tides, which can be of the order of $5{\cdot}10^{-7}{\cdot}g$, can be considered negligible [6].

In this way, we approximated the true gravity value g for a location at latitude φ and altitude H (height above mean sea level), by normal gravity γ_0 (4) and its corresponding decrease for altitude (3a) and atmospheric effect (3b),

$$g \approx \gamma_0 - C_H - A_H \tag{6}$$

References

1. Hofmann-Wellenhof, B.; Moritz, H. *Physical Geodesy*; Springer-Verlag: Vienna, 2005; ISBN 3-211-23584-1.
2. Kvas, A.; Brockmann, J.M.; Krauss, S.; Schubert, T.; Gruber, T.; Meyer, U.; Mayer-Gürr, T.; Schuh, W.D.; Jäggi, A.; Pail, R. GOCO06s- A Satellite-Only Global Gravity Field Model. *Earth Syst Sci Data* 2021, *13*, 99–118, doi:10.5194/ESSD-13-99-2021.
3. Moritz, H. Geodetic Reference System 1980. *Bulletin Géodésique* 1992, *66*, 187–192, doi:10.1007/BF00989270.
4. Hofmann-Wellenhof, B.; Moritz, H. *Physical Geodesy*; Springer-Verlag: Vienna, 2005; ISBN 3-211-23584-1.
5. Geospatial Sciences Division Computations. *NGA Gravity station data format and anomaly computations. St Louis, Mo* 1999.
6. Torge, W.; Müller, J.; Pail, R. Geodesy. *Geodesy* 2023, 1–506, doi:10.1515/9783110250008.
