# Supplementary material for: Enhanced Accuracy in Jump Power Estimation Using Photoelectric Cell System and GRS80 Location-Specific Gravitational Acceleration
Source: Sensors (Basel). 2025 Aug 20;25(16):5163. doi: 10.3390/s25165163 (PMC12389838; doi:10.3390/s25165163)
Supplement: Supplementary file 1 [file sensors-25-05163-s001.zip › Supplementary Materials File S2.pdf]

Supplementary Materials File S2:
Calculation of uncertainty associated with power measurement.

J.L. González-Montesinos [1], F.G. Montesinos[2,3], J.R. Fernández Santos[1], A. Suárez Llorens[4], I. Caraballo[1,*], P. Gutiérrez Mulas[5,], J.V. Gutiérrez-Manzanedo[1].

[1]Department of Physical Education, Faculty of Education Sciences, University of Cádiz (11510 Cádiz, Spain).
[2] Faculty of Mathematics Sciences, Complutense University of Madrid (28040 Madrid, Spain).
[3] Research Group "Geodesia", Complutense University of Madrid (28040 Madrid, Spain).
[4] Department of Statistics and Operation Research, University of Cádiz (11510 Cádiz, Spain).
[5] Faculty of Physical Activity and Sport Sciences. Complutense University of Madrid (28040 Madrid, Spain).

* Corresponding author: Israel Caraballo Vidal. e-mails: israel.caraballo@uca.es

Calculation of uncertainty associated with power measurement.

The uncertainty of a measurement is the doubt that exists regarding the quality of a measurement result. Being aware of the uncertainty of measurements is a fundamental concept because no further measurements should be taken if the uncertainty associated is unknown. During any measurements, a series of errors always appear, originating from different sources, such as the measuring instrument, environmental conditions, or the evaluator taking the measurements.

The calculation, in this case of muscle power using a kinematic system such as laser bars, is associated with a possible measurement error, i.e., its result does not exactly coincide with the true magnitude value; this is due to the error induced by the direct measurements involved in the calculation, namely, the flight and contact times and the gravity value (g). Evidently, the evaluation of this error is essential for subsequent comparison of the muscle power resulting from different geographical areas. As usual, we evaluated this error according to the procedure described by the international Guide to the Expression of Uncertainty in Measurement (GUM), which presents a homogeneous, rigorous, and unified treatment for its calculation [1]. Based on the GUM, we estimated the deviation of the measured value as regards the true value by linearizing the power expression and studying the propagation of error.

Based on the power expression given by the function P (g, t_v, and t_c), (Table 1) and denoting $m = (g^R, t_v^R, t_c^R)$, the 3-tuple formed by the true values of each magnitude, and P^R = f(m) the true power value, we obtain the following expression via the Taylor series of P^R:

$$P = f(g, t_v, t_c) = P^R + \alpha(g - g^R) + \beta(t - t_v^R) + \delta(t - t_c^R) + O(2^{nd} Order), \quad (1)$$

g: Earth's gravity value (m·s^{-2}).
t_v: Flight time of vertical jumps (s).
t_c: Contact time of vertical jumps (s).

$$\alpha = \left.\frac{\partial f}{\partial g}\right|_m = \left.\frac{2\text{穏?}_v ? t_v + t_c)}{4?t_c}\right|_m, \ \beta = \left.\frac{\partial f}{\partial t_v}\right|_m = \left.\frac{g^2 ?2? t_v + t_c)}{4?t_c}\right|_m, \text{ and } \delta = \left.\frac{\partial f}{\partial t_c}\right|_m = -\left.\frac{g^2 \cdot t_v^2}{4?t_c^2}\right|_m. \quad (2)$$

From the previous expression, Var(P), is given by:

$$Var(P) = \alpha^2 Var(g) + \beta^2 Var(t_v) + \gamma^2 Var(t_c) + 2\alpha\beta Cov(g, t_v) + 2\alpha\gamma Cov(g, t_c) + 2\beta\gamma Cov(t_v, t_c) \quad (3)$$

where Var(g), Var(t_v), and Var(t_c) correspond to the variance in the measurement of gravity, time of flight, and contact time, respectively. The terms $Cov(g, t_v)$, and $Cov(g, t_c) Cov(t_v, t_c)$ refer to covariance.

Table 1 S2. Coverage, deviation, and variance in the direct measurements.

Direct measurements	Coverage	Deviation	Variance
g	1 microgal $=10^{-8}$ m/s^2	$5{\cdot}10^{-9}$ m/s^2	$25{\cdot}10^{-18}$ m^2/s^4
$t_{v/jump}$	1 millisecond = 10^{-3} s	$5{\cdot}10^{-4}$ s	$25{\cdot}10^{-8}$ s^2
$t_{c/jump}$	1 millisecond = 10^{-3} s	$5{\cdot}10^{-4}$ s	$25{\cdot}10^{-8}$ s^2

By the very nature of the experiment, the error in the gravity measurement is independent of the error in the time measurements. On the other hand, the errors in both times are also independent. Therefore, the three covariance terms equal zero. At this point, it should be noted that the flight and contact times correspond to aggregate variables, where all flight and contact times obtained for the athlete's total number of jumps are added. In other words, if n represents the total number of jumps of an athlete in a given test and t_{v_i} and t_{c_i} the times of flight and contact in the ith jump, i = 1,...,n, we obtain:

$$t_v = \sum_{i=1}^{n} t_{v_i} \ y \ t_c = \sum_{i=1}^{n} t_{c_i}. \quad (4)$$

A direct check showed that there was no correlation between the different times associated with each jump, mainly due to the jump's short duration not incurring excessive fatigue. Then, from the expression of the variance of the sum of uncorrelated variables, we obtain the final expression of Var(P) equal to

$$Var(P) = \alpha^2 \cdot Var(g) + \beta^2 \cdot n \cdot Var(t_{v/jump}) + \gamma^2 \cdot n \cdot Var(t_{c/jump}), \quad (5)$$

where n represents the final number of jumps in the test. Finally, the variability in the previous measurements is given in Table 2.

Table 2 S2. Power and coverage for a hypothetical athlete represented by the average values of flight and contact time per jump, average number of jumps, and true gravity in Cádiz (Spain) (g = 9.79857796 m s^{-2}).

	Averages					
Test	t_v (s)	t_c (s)	n	Power (W)	Variance (W^2)	Coverage (W)
P_{ow15}	9.7143	4.9298	16.9	692.563781	0.09610808	0.62002606
P_{ow30}	19.5547	10.0193	35.4	1385.2837	0.19653098	0.8866363
P_{ow60}	38.1309	21.5848	74.9	2531.81771	0.32668256	1.14312302

As usual, estimates of the true value and variance, $\hat{P}^R$ and $\widehat{Var}(P)$, were obtained from the observed values. Finally, the coverage interval $\hat{P}^R \pm 2\sqrt{\widehat{Var}(P)}$ contained the true power value with 95.44% confidence.

As an example, Table 2 shows the power values estimated in Cádiz (Spain) (g = 9.79857796 m s^{-2}) as well as the coverage for the average values of flight and contact time and number of jumps in the three tests. It should be noted that, due to the greater number of jumps, the coverage error increases slightly with the duration of the test. This error was very small percentwise in all cases as regards the measurement value. As another example, Table 3 also shows the differences in power measurements using gravity GRS80 in Cádiz (g=9.79857796 m s^{-2}) versus the standard value (g = 9.81 m s^{-2}). We can again see from the coverage of the measurement that the difference observed—in this case, overestimated—was significant and increased as the duration of the test increased.

Table 3 S2. Difference in power and coverage for the power measurements in Cádiz (Spain) using the true gravity value and the standard value for a hypothetical athlete represented by the average values of time of flight and contact per jump and average number of jumps.

	Averages			Power Cádiz (g=9.798 ms^{-2})-Power Cádiz (g=9.81 ms^{-2})		
Test	t_v (s)	t_c (s)	n	Difference (W)	Variance (W^2)	Coverage (W)

P_{ow15}	9.7143	4.9298	16.9	-1.697459612	3.1965E-08	0.00035758
P_{ow30}	19.5547	10.0193	35.4	-3.395301916	6.53661E-08	0.00051134
P_{ow60}	38.1309	21.5848	74.9	-6.205433239	1.08658E-07	0.00065927

Thus, accuracy is conditioned by the value closest to reality, and these power values are estimated with a precision that is not altered by the uncertainty calculation associated with these measurements.

The calculation of the coverage of the difference between two power values associated with different regions follows the same procedure; in this case, the gravity variance increases because two measurements must be considered. In Table 4, we again demonstrate, as an example, the estimated values of the power difference between Cádiz (Spain) and Miami (USA), chosen due to having very similar gravity values. In Table 4, we show the difference for a hypothetical athlete, whose average values of flight and contact time and number of jumps in the three tests coincided in both cities, considering the true gravity values in Cádiz and Miami. As can be observed, even though the difference in gravity is minimal, the difference between the power values is significant; this becomes even clearer as the gravity values become more distanced. For example, to achieve the same result in P_{ow60}, there is a significantly lower power output of 4.6490905 W in Miami with respect to Cádiz. Evidently, if this test had a longer duration, the difference would be greater.

Table 4 S2. Difference in power and coverage in Miami and Cádiz for a hypothetical athlete represented by the average values of flight and contact time per jump and average number of jumps.

	Averages			Power Miami (g = 9.789 ms^{-2})-Power Cádiz (g = 9.798 ms^{-2})		
Test	t_v (s)	t_c (s)	n	Difference (W)	Variance (W^2)	Coverage (W)
P_{ow15}	9.7143	4.9298	16.9	-1.2717312	1.0071E-08	0.00020071
P_{ow30}	19.5547	10.0193	35.4	-2.5437492	2.0595E-08	0.00028702
P_{ow60}	38.1309	21.5848	74.9	-4.6490905	3.4238E-08	0.00037007

Therefore, none of the power measurements obtained, as well as hypothetical comparisons between regions with different gravity values, were altered by the calculation of the uncertainty associated with these measurements.

It was demonstrated that considering the approximate true gravity value entailed a significant difference in the power calculation compared with the use of the constant gravity value (9.81 m·s^{-2}) and that the precision estimate in this calculation was not altered by the calculation of the uncertainty associated with these measurements. We could therefore perform a comparative study of power variations in different cities from the jump sample.

References

1. Lequin, R.M. Guide to the Expression of Uncertainty of Measurement: Point/Counterpoint. *Clinical chemistry* **2004**, *50*, 977–978, doi:10.1373/clinchem.2003.030528.
